# Whole breast radiotherapy in prone and supine position: is there a place for multi-beam IMRT?

**DOI:** 10.1186/1748-717X-8-151

**Published:** 2013-06-24

**Authors:** Thomas Mulliez, Bruno Speleers, Indira Madani, Werner De Gersem, Liv Veldeman, Wilfried De Neve

**Affiliations:** 1 Department of Radiotherapy, Ghent University Hospital, De Pintelaan 185, Ghent 9000, Belgium; 2 Ghent University, De Pintelaan 185, Ghent 9000, Belgium

**Keywords:** Whole-breast irradiation, Prone position, Supine position, Wedged tangential fields, Intensity-modulated radiotherapy, Tangential field-IMRT, Multi-beam-IMRT

## Abstract

**Background:**

Early stage breast cancer patients are long-term survivors and finding techniques that may lower acute and late radiotherapy-induced toxicity is crucial. We compared dosimetry of wedged tangential fields (W-TF), tangential field intensity-modulated radiotherapy (TF-IMRT) and multi-beam IMRT (MB-IMRT) in prone and supine positions for whole-breast irradiation (WBI).

**Methods:**

MB-IMRT, TF-IMRT and W-TF treatment plans in prone and supine positions were generated for 18 unselected breast cancer patients. The median prescription dose to the optimized planning target volume (PTV_optim_) was 50 Gy in 25 fractions. Dose-volume parameters and indices of conformity were calculated for the PTV_optim_ and organs-at-risk.

**Results:**

Prone MB-IMRT achieved (p<0.01) the best dose homogeneity compared to WTF in the prone position and WTF and MB-IMRT in the supine position. Prone IMRT scored better for all dose indices. MB-IMRT lowered lung and heart dose (p<0.05) in supine position, however the lowest ipsilateral lung doses (p<0.001) were in prone position. In left-sided breast cancer patients population averages for heart sparing by radiation dose was better in prone position; though non-significant. For patients with a PTV_optim_ volume ≥600 cc heart dose was consistently lower in prone position; while for patients with smaller breasts heart dose metrics were comparable or worse compared to supine MB-IMRT. Doses to the contralateral breast were similar regardless of position or technique. Dosimetry of prone MB-IMRT and prone TF-IMRT differed slightly.

**Conclusions:**

MB-IMRT is the treatment of choice in supine position. Prone IMRT is superior to any supine treatment for right-sided breast cancer patients and left-sided breast cancer patients with larger breasts by obtaining better conformity indices, target dose distribution and sparing of the organs-at-risk. The influence of treatment techniques in prone position is less pronounced; moreover dosimetric differences between TF-IMRT and MB-IMRT are rather small.

## Background

Conventional radiotherapy (RT) using wedged tangential fields (W-TF) after breast-conserving surgery improves disease control and breast-cancer related survival. However prolonged follow-up showed an increased RT-induced risk of cardiac events and secondary lung and breast cancer in long-term survivors [1-3]. Therefore strategies for sparing organs-at-risk (OARs), while maintaining an adequate dose coverage of the target are warranted.

In supine position the whole-breast clinical target volume (CTV_WBI_) is concave *1)* enwrapping the lung and heart at the left side, and *2)* medially adjoining the contralateral breast. Therefore parts of the ipsilateral lung, heart, and contralateral breast may receive intermediate to high doses with W-TF.

Intensity-modulated radiotherapy (IMRT) can provide advantages compared to W-TF. In supine position IMRT using a tangential two-beam set-up (TF-IMRT) can improve dose homogeneity; however its ability to reduce high-dose regions to the underlying heart and lung tissue appear to be limited [4,5]. Supine multi-beam IMRT (MB-IMRT) may overcome those limitations often at cost of low- or intermediate-dose spread over the contralateral breast and ipsilateral thoracic region [6-10].

Prone position modifies the target volume by gravity and moves the breast away from the chest wall. Prone W-TF has previously been used for large, pendulous breasts [11] to reduce fibrosis and improve cosmesis [12,13]. There are a few studies reporting improved dosimetry by prone TF-IMRT [14-16], though data on whole-breast MB-IMRT in prone position are lacking. Moreover, all dosimetric studies comparing prone and supine position used only non-multi-beam techniques [16-20]. We performed the present study to establish the effect of treatment technique (W-TF, TF-IMRT or MB-IMRT) and position (prone or supine) on dose coverage and heart and lung sparing.

## Methods

Eighteen unselected early stage breast cancer patients - 6 right-sided and 12 left-sided - presenting for whole-breast irradiation (WBI) without nodal irradiation after breast conserving surgery were included in this study. Three-mm thick computer-tomography scans were acquired with an Aquilion scanner (Toshiba Medical Systems, Tokyo, Japan) in all patients in prone and supine position. Patient set-up and delineation of the clinical and planning target volumes for WBI (CTV_WBI_ and PTV_WBI_, respectively) and OARs in both treatment positions can be found elsewhere [16,17]. Extension of the PTV_WBI_ outside the skin into the air accounted for respiration-related breast movement or swelling of the breast during treatment. A flash region was created outside the patient’s external contour by expanding the PTV_WBI_ with a 10 mm margin followed by subtraction of the patient’s total scanned volume. This flash region was subsequently used in the optimization. A planning target volume for optimization (PTV_optim_), a structure used during plan optimization, was generated by removing the in-air part and a 7 mm-wide build-up region underneath the skin from the PTV_WBI_.

**Figure 1:**
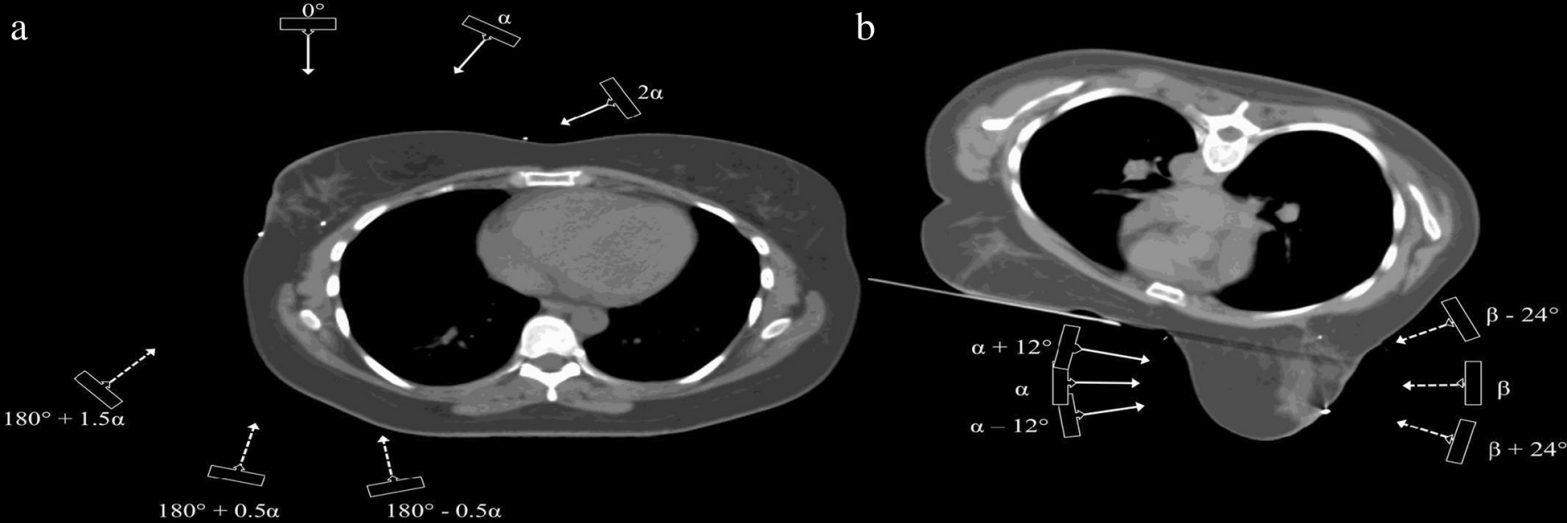
**Multi-beam set-up in the prone and supine position.** A 6-beam set-up used in the multi-beam intensity-modulated radiotherapy (MB-IMRT) plans for right-sided breast tumors in supine (**a**) and prone position (**b**). Gantry angles expressed in the Elekta coordinate system. The most inclined medial beam has the gantry angle of a tangential beam set by virtual simulation [21]. The gantry angles are 0°, |α|, |2α|, 180° - 0.5|α|, 180° + 0.5|α|, and 180° + 1.5|α| for supine MB-IMRT. The lateral gantry angles in prone MB-IMRT are |α|, |α|+/−24°, the medial gantry angles are |β|, |β|+/− 12°.

The dosimetric comparison was made for 6 MV photon beams of an Elekta SLi18 linear accelerator (Elekta, Crawley, UK) equipped with a standard 1 cm leaf-width multileaf collimator (MLC). A median prescription dose to the PTV_optim_ was 50 Gy in 25 fractions of 2.0 Gy with the objective of ≥95% of the PTV_optim_ receiving >95% of the prescribed dose and minimization of maximum dose, dose heterogeneity and “hot spots”. In both positions TF-IMRT used the same gantry angles as W-TF with the collimator set at 0° and the beams shaped around the PTV_WBI_ with the aid of the MLC. Figure 1 shows the 6-beam setup used in the MB-IMRT plans for right-sided breast tumors in supine position (a) and prone position (b). In both positions MB-IMRT used 6 coplanar beams shaped around the PTV_WBI_ and as in TF-IMRT plans field-in-field segments were created avoiding the ipsilateral lung, heart (in case of left-sided breast tumors) and contralateral breast (for lateral beams in supine position, since medial beams did not traverse the contralateral breast).

A forward planning approach was used for the intensity-modulated and W-TF plans. The convolution-superposition dose engine of a Pinnacle version 9.0 treatment planning system (Philips Medical Systems, Andover, US) was used for dose computations between optimization cycles of intensity-modulated plans as well as for final plans. Monitor units and MLC shapes were optimized using the optimization tools described before [21]. During optimization, two patient geometries were taken into account: 1) dose computation for PTV_WBI_ was performed using a density override (1 g/cm^3^) to the above-mentioned flash region; 2) dose computation for the PTV_WBI_ without build-up and OARs was performed without density overrides. To be able to compute both dose distributions in parallel, the patient data at the Pinnacle treatment planning system were duplicated: for the first patient dataset the flash region was set water-equivalent, while for the second patient dataset, the flash region remained at the density of the CT data (in essence, air outside the patient outline). To avoid hot spots outside regions of interest, a “matroska” sequence of shell structures [22] was generated outside the PTV_WBI_, which were taken into account during optimization. Dose computation for these shell structures was performed using the above-mentioned density override in the flash region. Also the dose update mechanism for changes in leaf positions during optimization took both patient geometries into account. This method was used mainly to account for substantial deformations of the breast during the course of treatments.

D_2_ and D_98_, or the dose exceeding 2% and 98% of the dose-volume histogram (DVH) points, respectively, were used as surrogates for maximum and minimum dose. These were evaluated for the PTV_optim_, as well as dose homogeneity (1-(D_2_-D_98_/median dose)). For the heart and ipsilateral lung D_2_, mean dose (D_mean_), V_5_, V_10_, V_20_ and V_25_ or the proportion of the volume receiving at least 5 Gy, 10 Gy, 20 Gy and 25Gy, respectively, were extracted from the DVH data. For the contralateral breast D_2_ and D_mean_ were evaluated.

The following indices were also calculated for the PTV_optim_:

Jaccardindex=A∩B/A∪B

Where A is the volume covered by the PTV_optim_ and B is the volume covered by the 95% isodose, i.e., the volume receiving 47.5 Gy or more. The Jaccard index increases with increase in similarity or overlap between the target volume and the 95% isodose and is a measure of dose conformity of the treatment plan.

Dose−coverageindex=A∩B1/A

Where B1 is the volume covered by the 95-107% isodose, i.e. the volume receiving between 47.5 Gy and 53.5 Gy. The dose-coverage index calculates the proportion of the target, in which the treatment-planning objectives for the target are met.

Mismatchindex=B2/B

Where B2 is the volume covered by the 95% isodose and lying outside the PTV_optim._. It is the fraction of the 95% isodose non-overlapping the target. If the mismatch index is large, large amounts of normal tissues receive 95% of the prescription dose, i.e., 47.5 Gy.

One-way analysis of variance (ANOVA) was used for a pairwise comparison of dose-volume parameters and indices between MB-IMRT, TF-IMRT and W-TF in the 2 treatment positions.

## Results

**Figure 2:**
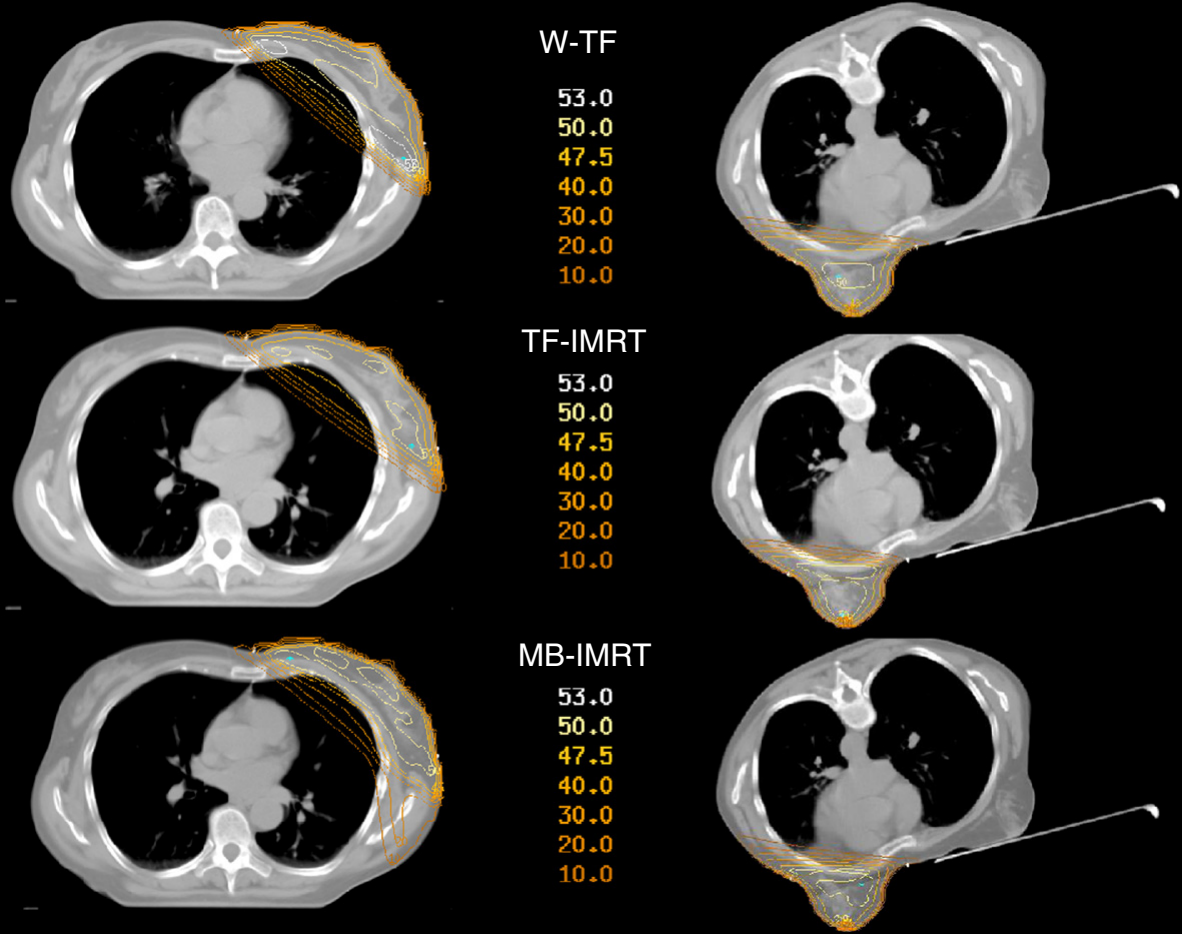
**Isodose distributions (in Gy) of the 6 treatment plans for a left-sided patient in a transverse plane. ***Abbreviations: W-TF = wedged tangential fields; TF-IMRT = tangential field intensity-modulated radiotherapy; MB-IMRT = multi-beam intensity-modulated radiotherapy.*

One hundred-and-eight plans were generated. Figure 2 illustrates typical dose distributions obtained with the 3 techniques in prone and supine position.

### Dose homogeneity and dose coverage of the target

Table 1 provides numerical data on target coverage and target dose distribution obtained with the 3 techniques in the prone and supine position. D_2_ is lowered in prone position resulting in improved dose homogeneity since D_98_ was similar for both positions. Significance was obtained for prone MB-IMRT versus all supine techniques and a trend (p=0.05) for prone TF-IMRT compared to supine W-TF regarding D_2_; moreover prone MB-IMRT obtained better (p<0.01) dose homogeneity compared to supine W-TF and MB-IMRT. Intensity-modulated techniques were able to improve dose homogeneity compared to conventional techniques in both positions, though significance (p=0.002) was only gained for prone MB-IMRT versus prone W-TF.

**Table 1:** **Dose-volume parameters (a) and conformity indices (b) for the optimized planning target volume (PTV**_**optim**_**)**

	**D**_**2**_**[Gy]**						**D**_**98**_**[Gy]**						**Dose homogeneity [%]**					
	**Prone**			**Supine**			**Prone**			**Supine**			**Prone**			**Supine**		
	**mean**	**SEM**	**SD**	**mean**	**SEM**	**SD**	**mean**	**SEM**	**SD**	**mean**	**SEM**	**SD**	**mean**	**SEM**	**SD**	**Mean**	**SEM**	**SD**
**(a)**																		
W-TF	52.3	0.1	0.6	53.1	0.2	0.9	47.6	<0.1	0.1	47.9	<0.1	0.4	90.6	0.3	1.1	89.7	0.5	2.1
TF-IMRT	52.0	0.2	0.8	52.6	0.2	0.8	47.8	<0.1	0.3	47.9	0.1	0.5	91.8	0.4	1.7	90.7	0.5	2.3
MB-IMRT	51.6	0.2	0.7	52.6	0.1	0.6	47.9	<0.1	0.2	47.7	<0.1	0.2	92.5	0.3	1.4	90.3	0.3	1.2
	**Jaccard index [%]**						**Dose-coverage index [%]**						**Mismatch index [%]**					
	**Prone**			**Supine**			**Prone**			**Supine**			**Prone**			**Supine**		
	**mean**	**SEM**	**SD**	**mean**	**SEM**	**SD**	**mean**	**SEM**	**SD**	**mean**	**SEM**	**SD**	**mean**	**SEM**	**SD**	**mean**	**SEM**	**SD**
**(b)**																		
W-TF	74.9	2.0	8.5	52.9	3.6	15.2	97.2	0.2	1.0	96.2	0.6	2.4	23.9	2.0	8.6	46.8	3.6	15.4
TF-IMRT	74.8	1.5	6.2	64.6	2.1	8.9	97.7	0.2	0.8	96.6	0.3	1.2	24.4	1.5	6.4	34.7	2.1	9.1
MB-IMRT	77.1	1.4	5.9	70.5	1.6	6.7	97.8	0.1	0.6	96.5	0.2	1.0	22.1	1.4	6.0	28.5	1.6	6.8

*Abbreviations: SEM* Standard error of the mean, *SD* Standard deviation, *W-TF* Wedged tangential fields, *TF-IMRT* Tangential field intensity-modulated radiation therapy, *MB-IMRT* Multi-beam intensity-modulated radiation therapy.

Prone WBI scored better for Jaccard and mismatch indices (Table 1). Prone MB-IMRT achieved better results than any supine treatment technique (p≤0.03, both indices); followed by prone TF-IMRT versus supine TF-IMRT and W-TF (p≤0.001, both indices). In supine position MB-IMRT (p<0.001) was the best and W-TF (p<0.001) was the worst technique for both indices. Prone IMRT improved significantly (p<0.01) dose coverage index: prone TF-IMRT vs. supine MB-IMRT and prone MB-IMRT vs. supine MB-IMRT and TF-IMRT.

### Dose-volume parameters in OARs

**Figure 3:**
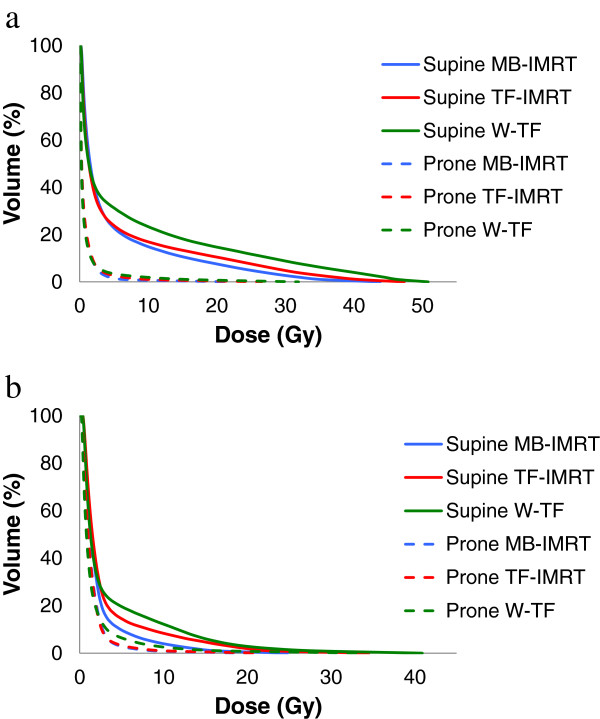
**Cumulative dose-volume histograms of the ipsilateral lung (a) and heart (b).** All patients were included for the ipsilateral lung, while for the heart only left-sided breast cancer patients were evaluated. *Abbreviations: W-TF = wedged tangential fields, TF-IMRT = tangential field intensity-modulated radiotherapy, MB-IMRT = multi-beam intensity-modulated radiotherapy.*

Figure 3 illustrates cumulative DVHs of the ipsilateral lung (all patients) and heart (only left-sided patients), numerical data are presented in Table 2. Sparing (p<0.001) of the ipsilateral lung by radiation dose was always superior in prone. There was little difference in ipsilateral lung dose between the 3 techniques in prone position, although V_10_ and V_20_ were significantly lower in prone MB-IMRT vs. prone W-TF. In supine position treatment technique did alter lung dose (p<0.05), MB-IMRT achieved the best and W-TF the worst lung avoidance by radiation dose. A remarking feature is the modified (p=0.003) ipsilateral lung volume in both positions. Mean ± standard deviation for ipsilateral lung volume is 1504 ± 401cc for prone position versus 1409 ± 431cc for supine position.

**Table 2:** Mean ± standard deviation for ipsilateral lung (all patients) and heart (only left-sided patients) dose metrics

**Technique**			**Ipsilateral lung**						**Heart**			
	**D**_**mean**_**[Gy]**		**V**_**20**_**[%]**		**V**_**25**_**[%]**		**D**_**mean**_**[Gy]**		**V**_**20**_**[%]**		**V**_**25**_**[%]**	
	**Prone**	**Supine**	**Prone**	**Supine**	**Prone**	**Supine**	**Prone**	**Supine**	**Prone**	**Supine**	**Prone**	**Supine**
W-TF	*1.2±0.6*	*7.7±4.5*	*0.9±1.0*	*13.5±10.2*	*0.7±0.8*	*12.1±9.3*	*1.9±1.1*	*3.9±3.4*	*1.2±0.6*	*4.9±1.9*	*0.8±1.7*	*4.0±5.8*
TF-IMRT	*1.1±0.5*	*5.7±3.1*	*0.5±0.7*	*9.8±7.0*	*0.3±0.5*	*8.5±6.4*	*1.6±0.5*	*3.3±2.5*	*0.4±0.2*	*3.4±1.4*	*0.3±0.6*	*2.9±4.3*
MB-IMRT	0.9±0.4	*5.1±2.6*	0.2±0.4	*7.6±6.2*	0.1±0.3	*6.4±5.5*	1.6±0.4	*2.5±1.7*	0.3±0.1	*1.9±0.9*	0.2±0.3	*1.4±2.2*

Abbreviations: *D*_*mean*_ Mean dose, *V*_*20*_*and V*_*25*_ Partial volume receiving at least 20 Gy and 25 Gy, respectively, *W-TF* Wedged tangential fields, *TF-IMRT* Tangential field intensity-modulated radiation therapy, *MB-IMRT* Multi-beam intensity-modulated radiation therapy.

Heart dose was lowered with MB-IMRT compared to TF-IMRT (D_2_, D_mean_, V_5;_ p=0.07, 0.05 and 0.03, respectively) and W-TF (D_2_, V_5_, p= 0.009 and 0.07, respectively) in supine position. While in prone position the effect of treatment technique on heart dose is less pronounced. Population averages for heart dose metrics were non-significantly lowered in prone compared to supine position. Better heart sparing by radiation dose was consistently obtained in prone position for patients with a PTV_optim_ volume >600cc. While for patients with a PTV_optim_ volume <600cc heart dose metrics were comparable (2/5 patients) or worse (3/5 patients) in prone position compared to supine MB-IMRT.

Neither treatment technique, nor set-up significantly changed doses in the contralateral breast, all procedures achieved a maximum dose <5Gy and mean dose <1.5Gy for all patients.

## Discussion

In supine position IMRT techniques obtain a higher Jaccard index, i.e. superior dose conformity, and less mismatch compared to W-TF with MB-IMRT being the superior technique for both indices. Dose conformity, coverage and mismatch are even better for the prone techniques, becoming statistically significant in prone IMRT plans. This is not surprising, since prone position results in a less concave breast volume. Therefore dose to the axillary and shoulder region is substantially reduced and less of the prescription dose can be expected to be out of the target. Our results confirm the reduction of dose inhomogeneity, with IMRT-techniques compared to standard W-TF. Though differences were rather small and non-significant in supine position, which could be explained by the use of non-mixture beam energies. Prone as compared to supine IMRT does improve dose homogeneity and hot spots with the best results in prone MB-IMRT plans. Our results are in agreement with other publications on prone IMRT. Goodman et al. [15] demonstrated a maximum dose in the target exceeding 110% with prone W-TF in 16 of 20 patients as compared to 1 patient with prone IMRT (TF-IMRT). Another study comparing MB-IMRT, TF-IMRT and 3D-CRT treatment plans of 5 patients planned in prone position reported significantly higher dose homogeneity of MB-IMRT plans vs. TF-IMRT (p=0.003) and 3D-CRT plans (p=0.03) [23]. Hardee et al. [14] observed a maximum dose reduction and improved median dose homogeneity in a prone TF-IMRT vs. 3D-CRT patient cohort. Moreover a 9%-decrease of grade 2 dermatitis and a 16%-reduction of grade ≥2 hyperpigmentation were found in the IMRT group. We expect that improved dose homogeneity and hot spots achieved by prone IMRT – either MB-IMRT or TF-IMRT - will yield lower skin toxicity and better cosmesis [4,5,24].

Lung irradiation was lowered with the MB-IMRT technique in supine position, though sparing of the ipsilateral lung appeared to be depending more on the treatment position than on the treatment technique. Prone position resulted in a spectacular decrease in lung dose, which is in coherence with other data [16-20]. The decrease in lung dose in prone position might also be attributed by the 7% increase in ipsilateral lung volume, for which we don’t have an explanation. All prone treatment techniques showed similar lung dose metrics.

Left-sided breast cancer patients are at risk of radiation-induced cardiac events [2], emphasizing the importance of using more sophisticated techniques to lower the heart dose. In supine position, MB-IMRT is able to lower the heart dose compared to the other techniques as shown both in our data and in other publications [7-9]. In prone position different treatment techniques have less effect on heart dose, especially between IMRT-techniques. Even with MB-IMRT, only the minority of patients (3/12) benefitted from supine position; which is in coherence with other data [18,20]. Moreover consistent better heart dose metrics were achieved in prone position for patients with a PTV_optim_ volume of >600cc. A limitation of this study is the absence of dose parameters of the left descending coronary artery, since this is likely associated with increased cardiac mortality.

The introduction of supine MB-IMRT was not successful because of its complexity, increase in dose to the contralateral breast and higher integral dose [7-9]. In contrast with these studies we selected beams that avoided the contralateral breast and removed beams that included too much lung tissue. In this way reducing the dose in the ipsilateral lung with MB-IMRT, both in supine and prone position, was not at cost of low-dose spread over the lung or heart as illustrated by the DVHs (Figure 3). The dose to the contralateral breast was not increased with MB-IMRT either, moreover a maximum dose <5Gy and mean dose <1.5Gy was obtained for all patients.

As a consequence of the reduced ipsilateral lung and heart dose, better dose distribution and dose coverage, prone IMRT is superior to any supine technique for left-sided patients with larger breasts (PTV_optim_>600cc) and all right-sided patients. While for left-sided patients with smaller breasts individual comparative planning should be made between supine MB-IMRT and prone IMRT in order to choose the best technique for clinical execution. The dosimetric differences between prone TF-IMRT and prone MB-IMRT are rather small. Whether these “small” dosimetric benefits would cause a clinical benefit is unknown. The more complex and time consuming planning procedure and beam delivery of prone MB-IMRT should also be considered.

## Conclusions

MB-IMRT is the preferred technique in supine position by providing better coverage indices of the target and sparing of organs-at-risk. However, prone IMRT is superior to any supine technique for right-sided breast cancer patients and left-sided breast cancer patients with larger breasts. The impact of treatment techniques in prone position is less prominent; moreover dosimetric differences between both IMRT-techniques are rather small.

## Abbreviations

RT: Radiotherapy; WBI: Whole breast irradiation; OARs: Organs-at-risk; W-TF: Wedged tangential fields; IMRT: Intensity-modulated radiotherapy; TF-IMRT: Tangential field intensity-modulated radiotherapy; MB-IMRT: Multi-beam intensity-modulated radiotherapy; CTVWBI: Whole-breast clinical target volume; PTVWBI: Whole-breast planning target volume; PTVoptim: Planning target volume for optimization; DVH: Dose-volume histogram; Dmean: Mean dose; D2 and D98: Dose exceeding 2% and 98% of the DVH points, respectively; V5,V10 , V20 and V25: Partial volume receiving at least 5 Gy, 10 Gy, 20Gy and 25 Gy, respectively; ANOVA: Analysis of variance.

## Competing interests

The authors declare that they have no competing interests.

## Authors’ contributions

IM, WDG, LV and WDN participated in the design and coordination of the study and helped to draft the manuscript. TM and BS conceived of the study, participated in the design and coordination of the study, participated in the treatment planning, carried out the dose calculations, performed the statistical analysis and drafted the manuscript. All authors read and approved the final manuscript.
